# The Effect of the Acetone Extract of *Arctotis arctotoides* (Asteraceae) on the Growth and Ultrastructure of Some Opportunistic Fungi Associated with HIV/AIDS

**DOI:** 10.3390/ijms12129226

**Published:** 2011-12-09

**Authors:** Wilfred M. Otang, Donald S. Grierson, Roland N. Ndip

**Affiliations:** 1 School of Biological and Environmental Sciences, Faculty of Science and Agriculture, University of Fort Hare, P/Bag X1314, Alice 5700, South Africa; E-Mails: wilfredotang5@yahoo.com (W.M.O.); RNdip@ufh.ac.za (R.N.N.); 2 Department of Biochemistry and Microbiology, Faculty of Science, University of Buea, Buea Box 63, Cameroon

**Keywords:** HIV/AIDS, opportunistic fungi, *Arctotis arctotoides*, scanning electron microscopy, energy dispersive X-ray spectroscopy

## Abstract

In this study, the effect of the acetone extract of *Arctotis arctotoides* (L.f.) O. Hoffm. (Asteraceae) on the growth and ultrastructure of some opportunistic fungi associated with HIV/AIDS was analyzed by means of scanning electron microscope (SEM). Remarkable morphological alterations in the fungal mycelia which were attributed to the loss of cell wall strength ranged from loss of turgidity and uniformity, collapse of entire hyphae to evident destruction of the hyphae. The elements responsible for giving the fungi their characteristic virulence were detected and quantified by energy dispersive X-ray microanalysis techniques. X-ray microanalysis showed the specific spectra of sodium, potassium and sulfur as the principal intersection of the four pathogenic fungi studied. Since these ions have the potential of fostering fungal invasion by altering the permeability of hosts’ membranes, their presence was considered inherent to the pathogenicity of the opportunistic fungi. Hence, these findings indicate the potential of the crude extract of *A. arctotoides* in preventing fungal invasion and subsequent infection of host’s membranes.

## 1. Introduction

The advent of HIV infection and immunosuppression induced for organ transplants or by cancer chemotherapy has led to increased predisposition to fungal infections [1]. A predominant source of morbidity and mortality among HIV positive individuals in late stages of HIV infection and low CD4 count (<500/mm^3^), is opportunistic infection caused by agents that rarely infect immuno competent individuals [2]. During the past several decades, there has been a steady increase in the frequency of opportunistic fungal infections (OFIs) such as candidiasis, aspergillosis and cryptococcal meningitis in immunocompromised patients [3]. Infections with *Candida albicans* appear when CD4 count is between 200–500/mm^3^ and may be the first indication of immunodeficiency [2]. *C. albicans* is also part of the normal oral flora and it usually grows as a harmless commensal. However, when local or systemic host defense mechanisms are impaired, this organism can proliferate and cause debilitating oropharyngeal candidiasis [4]. Invasive aspergillosis is a major cause of mortality in immunosuppressed patients [5]. *A. fumigatus* is responsible for more than 90% of invasive aspergillosis, with *A. flavus*, *A. terreus*, and *A. niger* responsible for the majority of the remaining infections [6]. Without effective host defenses following pulmonary exposure, the conidia resting in alveoli begin to enlarge and germinate [5]. Dermatophyte infection of the skin and nails in most HIV-infected patients is caused by species of the genera: *Epidermophyton*, *Microsporum* and *Trichophyton*. There is a lack of epidemiological data on dermatophyte infections because of their low morbidity, but it appears that HIV-positive patients have an incidence similar to that of HIV-negative patients [7]. The exception to this is proximal subungual onychomycosis, which occurs almost exclusively in immunocompromised patients.

Although new synthetic antifungal drugs have been successfully commercialized in recent years, they encountered major problems not only in regard to the adverse side effects on mammalian systems but also in the development of resistance by bacterial and fungal pathogens. Because of the clear tendency towards the optimization of alternative methods for disease control that produce minimal damage to the environment and human health [8], plant extracts, as one of the alternatives have been of much interest in diverse areas partly due to their affordability and unmatched chemical diversity. A holistic concept of the mode of action of plant-derived antifungal agent demands a proper understanding of both the chemical nature of the antifungal ingredient present and the structural and biochemical properties of the pathogenic fungi.

In our previous study [9], we investigated the antifungal activity of the hexane and acetone extracts of *Arctotis arctotoides* against some opportunistic fungi associated with HIV/AIDS. The most susceptible fungi, based on the overall mean diameter of growth inhibition were *Candida glabrata*, *C. krusei* and *Microsporum canis*, while *Cyptococcus neoformans*, *Trycophyton tonsurans* and *M. gypseum* were not susceptible to any of the extracts even at 5 mg/mL which was the highest concentration used. Many studies in ethnopharmacology have investigated the biological activities of various plant extracts and the chemical structures of many plant-derived antifungal compounds have been elucidated [8]. However, very little has been documented on the chemical and structural parameters which are responsible for the virulence of the fungi. Therefore, in this study, the effect of *A. arctotoides* (Asteraceae) acetone extract on the growth and ultrastructure of some opportunistic fungi associated with HIV/AIDS was analyzed by means of scanning electron microscopy (SEM). The elements responsible for giving the fungi their characteristic virulence were detected and quantified by energy dispersive X-ray microanalysis techniques. The result of this study will shed more light on the mode of action of natural products in general; which is a crucial factor in the development of new antifungal agents amidst the obvious dearth of effective and safe antifungal drugs.

## 2. Results

### 2.1. Inhibition of Fungal Growth

Results from the exposure of the treated fungi at various concentrations of the acetone extract of *A. arctototides* at 37 °C in SDB are shown in Table 1. The inhibition of mycelia growth ranged from 0% in *A. fumigatus* at a concentration of 0.32 mg/mL of plant extract to 65.3% in *C. albicans* at the highest concentration of 5 mg/mL. The plant extract inhibited mycelia growth in *C. albicans* and *M. canis* in a dose-dependednt manner. For *A. fumigatus*, inhibition of mycelia growth was observed only at 5 mg/mL, which was the highest concentration of extract used.

### 2.2. Results of SEM

**Figure 1:**
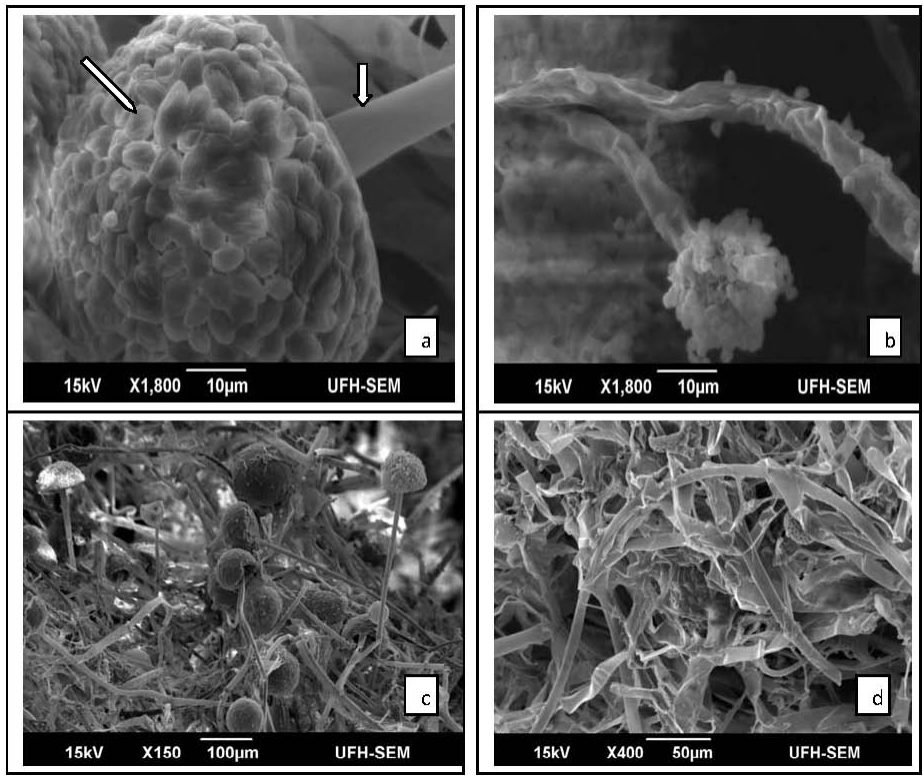
Scanning electron micrographs: (**a**) Conidium and conidiophore (arrows) of *M. canis* appear smooth and round (control), (**b**) conidia disintegrates and conidiophores shrinks in 5 mg/mL of plant extract; (**c**) pseudohyphae of *C. albicans* (control), (**d**) note breakage and deformation of entire mycelia when treated.

**Figure 2:**
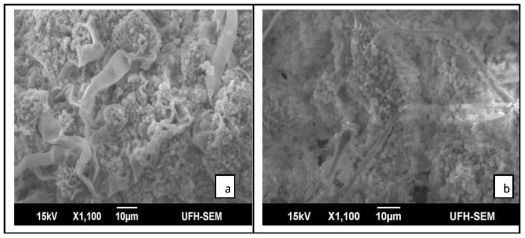

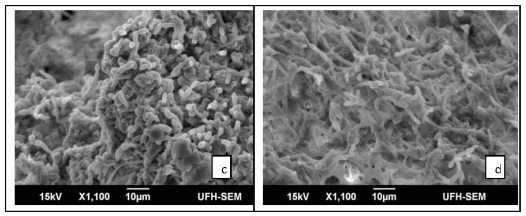
Scanning electron micrographs: (**a**) Conidiophore and conidia of *A. fumigatus*, (**b**) note massive collapse of conidia when treated with plant extract; (**c**) yeast of *C. glabrata*, (**d**) treated yeast cells appear flaccid, distended and rough.

The study showed variability in the ultrastructure of the fungi grown in medium supplemented with the plant extract as compared to the control. Electron microscopic observations revealed that untreated *M. canis* conidiophore displayed complete tubular shape of approximately 6 μm in diameter (Figure 1a). After being exposed to the plant extract of 5 mg/mL, the conidiophore showed aberrant morphologies including shrinkage and partial distortion with reduced diameter of approximately 4 μm (Figure 1b). Deformation of conidia was also observed in treated *M. canis* (Figure 1b). Remarkable morphological alterations were visible in treated *C. albicans*; including deformation of the mycelia, pseudohyphae appeared flattened and distorted (Figure 1c) while the pseudohyphae of the control appeared clear, smooth and turgid (Figure 1d). *A. fumigatus* grown on SDB (control) had normal hyphae and conidiophores (Figure 2a), while the fungus exposed to 5 mg/mL of plant extract showed disintegration of whole conidia (Figure 2b). A normal budding profile was observed in *C. glabrata* (Figure 2c); yeast cells appeared smooth and turgid, while yeast cells of treated *C. glabrata* appeared distended, flaccid and rough (Figure 2d). No significant morphological changes were observed in the fungal mycelia that were treated with lower concentrations (<5 mg/mL) of the plant extract, when compared to the control.

### 2.3. Results of EDXS

**Figure 3:**
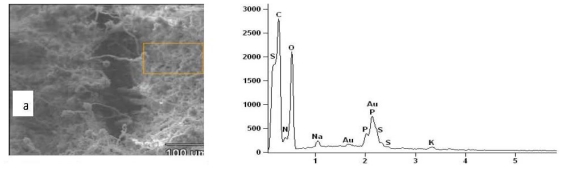

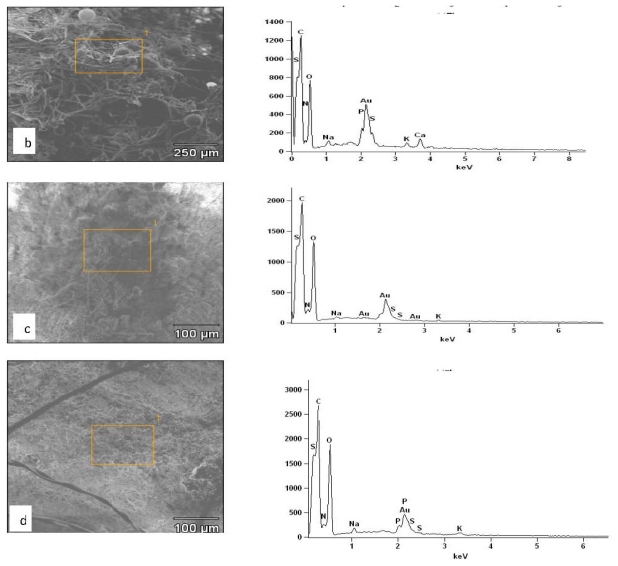
Characteristic X-ray spectra of various elements detected in the opportunistic fungi: (**a**) *M. canis*; (**b**) *C. albicans*; (**c**) *A fumigatus*; (**d**) *C. glabrata*. Micrographs on the left of each spectrum show the point of focus of the electron beam (anticathode).

**Figure 4:**
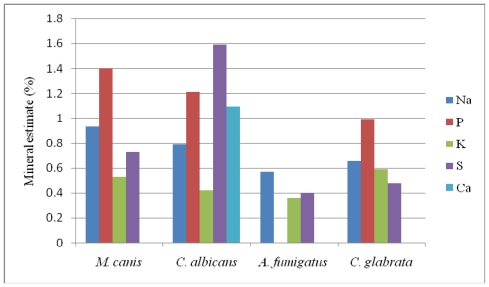
Quantitative estimates of various elements detected in the opportunistic fungi by EDXS.

X-ray microanalysis of *M. canis*, *C. albicans* and *C. glabrata* showed the specific spectra of the following elements: calcium (Ca), potassium (K), sulfur (S), phosphorus (P) and sodium (Na) on the PET crystal detector (Figure 3a–d). The spectrum of calcium was detected only in *C. albicans* (Figure 3b) and phosphorus was not detected in the microanalysis of *A. fumigatus* (Figure 3c). The natural mineral contents found in living tissues, namely, carbon, hydrogen, oxygen, and nitrogen were expected, while gold (AU) was assumed to be derived from the spur coater. The peak heights of the elements in the spectra show their comparative measures while the quantitative estimates of their absolute concentrations are shown in Figure 4. The common characteristic of all the four pathogenic fungi was the presence of sodium, potassium and sulfur. *C. albicans* and *A. fumigatus* were characterized by the richness of sulfur and the complete absence of phosphorous respectively, as opposed to rich levels of potassium in both *M. canis* and *C. glabrata* (Figure 4).

## 3. Discussion

In the present study, the acetone extract of *A. arctotoides* has shown fungistatic activity against the tested fungi when compared to the control, except at lower extract concentrations against *A. fumigatus. A. arctotoides* is a decumbent herb commonly found as roadside weed in most coastal districts of South Africa [10]. The *Xhosa*-speaking people in the Eastern Cape Province apply the juice from the leaf as a topical paste to treat wounds [11]. The inhibitory activity of the aqueous extracts of the shoots of *A. arctotoides* against six fungal species, with growth inhibitions ranging from 50.7% on *Aspergillus tamarii* to 95.2% on *Penicillium digitatum* at 0.1 mg/mL has also been reported [12]. The acetone and methanol extracts of the root of this plant demonstrated significant activity against five fungal species: *Aspergillus flavus*, *A. niger*, *Fusarium oxysporium*, *Mucor heamalis*, and *Penicillium notatum.* Inhibition of fungal growth ranged from 56.23% on *A. flavus* to 100% on *P. notatum* and *M. heamalis* at 5.0 mg/mL [10].

However, to the best of our knowledge, there are no reports on the effect of *A. arctotoides* extract on fungal ultrastructure. Hence, we seem to report here for the first time the effects of the acetone extract of *A. arctotoides* in relation to ultrastructural alterations in fungal compartments. Ultrastructural changes of fungal compartments have likewise been reported for *A. niger* hyphae treated with *Cympobogon nardus* [13]. The observed alterations in the fungal ultrastructure could be attributed to the impact of the plant extract on the cell wall with the resultant changes in the fungal hyphae. These changes may be related to the loss of cell wall strength, which is responsible for the strength and integrity of the cell and thus determined the cell shape [14]. Fungal cells are supported by an internal hydrostatic pressure acting on the cell wall, rather than by an internal proteinaceous cytoskeleton as with animal cells, although a comparable cytoskeleton is essential for cell function [15]. Irreversible morphological changes in the cell wall and plasma membrane of *A. flavus* caused by *Ageratum conyzoides* extract have been reported [16]. de billerbeck *et al.* [17] reported a decrease in the cell wall thickness of *A. niger* caused by the essential oil of *Cymbopogon nardus*. These suggest that the cell wall could be one of the target sites of action of plant extracts. However, whether these changes are associated with direct interaction between the components of the plant extract with those of the cell wall, or are derived from interference with processes involved in cell wall synthesis remains elusive and therefore demands further investigation.

Chemical analysis with the EDXS is based on the principle that each element of the periodic system has a unique and well-defined characteristic X-ray spectrum. It seems that during their development, pathogenic fungi secrete extracellular material that includes the elements (Si, P, S, K, and Ca) that give them their characteristic virulence [18]. These ions may enhance their pathogenicity by affecting the permeability and subsequent invasion of hosts’ membranes. The presence of extracellular materials is not an uncommon feature of microorganisms [19]. A change in cell permeability might result in an imbalance in intracellular osmotic pressure, subsequent disruption of intracellular organelles, leakage of cytoplasmic contents and finally cell death [20]. Breaking down of the cell wall, degradation of cell organelles, and changes in cell permeability have been noted to occur as the most significant cellular alterations [21].

## 4. Experimental Section

### 4.1. Plant Material and Culture Conditions

The acetone extract of *A. arctotoides* leaves was prepared as previously described [4]. Sabouraud dextrose broth (SBD) was prepared according to the manufacturer’s prescription. The SDB medium was divided into 5 mL aliquots in test tubes and sterilized by autoclaving at 120 °C for 15 min. Different amounts of plant extracts were added to the test tubes to make final two-fold serial concentrations of 5–0.032 mg/mL. Test tubes containing only SDB were considered as controls. Spore suspensions (approximately 10^4^ spores/mL) of Candida *albicans* ATCC 2091, *C. glabrata* ATCC 2001, *Aspergillus fumigatus* ATCC 204305, and *Microsporum canis* ATCC 36299 prepared from 10-day cultures on sabouraud dextrose agar slants were aseptically transferred into the test tubes [12]. The tubes were incubated at 37 °C for various times depending on the fungi: 48 h for *C. albicans* and *C. glabrata*, 96 h for *A. fumigatus* and 6 days for *M. canis.* The experiment was repeated in duplicate.

### 4.2. Inhibition of Fungal Growth

Fungal mycelia were separated from culture media by passing through Whatman filter No. 1. A known amount of thoroughly washed mycelia was placed on preweighted petri plates and allowed to dry at 60 °C for 6 h to reach a constant weight [21]. Inhibition of mycelia growth was calculated according to the formula given below [22].

$$\begin{array}{c} \%\text{Mycelial inhibition}=[(\text{Mycellial}\;\text{growth }(\text{control})-\text{Mycellial growth}\\ (\text{treatment}))/(\text{Mycellial growth }(\text{control}))]\times 100. \end{array}$$

Statistically significant differences among the percentage inhibition values of fungal growth induced by the different concentrations of the plants extracts when compared with the control culture of each fungus were determined by one way analysis of variance (ANOVA) while the treatment means were compared using the Duncan’s multiple range test. All analyses were done with Minitab statistical software (student version 12 for windows).

### 4.3. SEM and Energy Dispersive X-ray Spectroscopy (SEM-EDXS)

Mycelia selectively obtained after exposure to various concentrations of the plant extract were fixed with glutaraldehyde (6%), critical point dried, mounted on graphite stubs and coated with gold [18]. Spectra and micrographs were obtained with a scanning electron microscope equipped with two wavelength spectrometers working with an accelerating voltage of 15 kV. Different regions of the mycelia were microanalyzed and the representative spectra are presented (see results). A focused beam of electrons was used to scan the fungal hyphae at the point where examination of its chemical composition was desired. The detection and determination of elements with the EDXS was based on the emission of characteristic X-rays bv the hyphae under bombardment with electrons. The dispersed spectra produce a pattern of X-rays characteristic of the element excited. Only the most intense emissions, the so-called K′ and Kα lines, were analyzed with the spectrometers [18].

## 5. Conclusion

The opportunistic fungi: *M. canis*, *C. albicans*, *C. glabrata* and *A. fumigatus* were analyzed by combining scanning electron microscopy with energy dispersive X-ray analysis. Remarkable morphological alterations were visible in these fungi when treated with the acetone extract of *A. arctotoides*. These changes were attributed to the loss of cell wall strength, which led to the subsequent alteration in fungal morphology. X-ray microanalysis showed the specific spectra of sodium, phosphorus and sulfur as the principal intersection of the four pathogenic fungi. Since these ions have the potential of fostering fungal invasion by altering the permeability of hosts’ membranes, their presence was considered inherent to the pathogenicity of the opportunistic fungi.

## Figures and Tables

**Table 1:** Inhibition of fungal growth by the acetone extract of *A. arctotoides*.

Concentration of plant extract (mg/mL)	Inhibition of mycelial growth (%) a			

	Af	Cg	Ca	Mc
0.32	0	14.3	9.4	15.6
0.63	0	19.5	20.7	26.3
1.25	0	14.3	25.3 *	29 *
2.5	0	29 *	41.8 *	41.4 *
5	9.7	25 *	65.3 *	59.5 *

a Values are means of mycelial growth inhibition. The control showed 0% inhibition for all fungal species. Ca *= C. albicans*, Cg = *C. glabrata*, Af *= A. fumigatus*, Mc =*M. canis*, control = SDB. Total fungal growth of control cultures (g): Ca = 0.52 ± 0.2, Cg = 0.66 ± 0.4, Af = 2.3 ± 1, Mc = 0.51 ± 0.4.

* Inhibition of mycelial growth is significantly lower than in the control culture at *P* < 0.05.

## References

[1] Walsh T.J., Groll A., Hiemenz J., Fleming R., Roilides E., Anaissie E. (2004). Infections due to emerging and uncommon medically important fungal pathogens. Clin. Microbiol. Infect.

[2] Bharathi M., Rani A.U. (2011). Pathogenic fungal isolates in sputum of HIV positive patients. J. AIDS HIV Res.

[3] Miceli H.M., Diaz J.A, Lee D.S. (2011). Emerging opportunistic yeast infections. Lancet Infect. Dis..

[4] Sun J.N., Solis N.V., Phan Q.T., Bajwa J.S., Kashleva H., Thompson A., Liu Y., Dongari-Bagtzoglou A., Edgerton M., Filler S.G. (2010). Host cell invasion and virulence mediated by *Candida albicans*. PLoS Pathog.

[5] Baddley J.W. (2011). Clinical risk factors for invasive aspergillosis. Med. Mycol.

[6] Thompson G.R., Patterson T.F. (2008). Pulmonary aspergillosis. Semin. Respir Crit. Care Med.

[7] Rodwell G.E., Bayles C.L., Towersey L., Aly R. (2008). The prevalence of dermatophyte infection in patients infected with human immunodeficiency virus. Int. J. Dermatol.

[8] Alvarez-Castellanos P.P., Bishop C.D., Pascual-Villalobos M.J. (2001). Antifungal activity of the essential oil of flowerheads of garland chrysanthemum (*Chrysanthemum coronarium*) against agricultural pathogens. Phytochemistry.

[9] Otang W.M., Grierson D.S., Ndip R.N. (2011). Antifungal activity of *Arctotis arctotoides* (L.f.) O. Hoffm. and *Gasteria bicolor* Haw. against opportunistic fungi associated with HIV/AIDS. Pharmacog. Mag.

[10] Afolayan A.J., Jimoh F.O., Sofidiya M.O., Koduru S., Lewu F.B. (2007). Medicinal potential of the root of *Arctotis arctotoides*. Pharm. Biol.

[11] Afolayan A.J. (2003). Extracts from the shoots of *Arctotis arctotoides* inhibit the growth of bacteria and fungi. Pharm. Biol.

[12] Salama H.M.H., Marraiki N. (2009). Antimicrobial activity and phytochemical analysis of *Polygonum Aviculare* L. (Polygonaceae), naturally growing in Egypt. Aust. J. Basic Appl. Sci.

[13] Sharma N., Tripathi A. (2008). Effect of *Citrus sinensis* (L.) Osbeck epicarp essential oil on growth and morphogenesis of *Aspergillus niger* (L.) Van Tieghem. Microbiol. Res.

[14] Gandomi H., Misaghi A., Basti A.A., Hamedi H., Shirvani Z.R. (2009). Effect of *Zataria multiflora* Boiss. Essential oil on growth and aflatoxin formation by *Aspergillus flavus* in culture media and cheese. Food Chem. Toxicol.

[15] Kaminskyj S.G., Dahms T.S. (2008). High spatial resolution surface imaging and analysis of fungal cells using SEM and AFM. Micron.

[16] Nogueira J.H.C., Goncalez E., Galleti S.R., Facanali R., Marques M.O.M., Felício J.D. (2010). *Ageratum conyzoides* essential oil as aflatoxin suppressor of *Aspergillus flavus*. Int. J. Food Microbiol.

[17] de Billerbeck V.G., Roques C.G., Bessiere J.M., Fonvielle J.L., Dargent R. (2001). Effect of *Cymbopogon nardus* (L.) W. Watson essential oil on the growth and morphogenesis of *Aspergillus niger*. Can. J. Microbiol.

[18] Figueras M.J., Guarro J. (1997). X-ray microanalysis of black piedra. Antonie van Leeuwenhoek.

[19] Sutherland I. (1989). Microbial polysaccharides—A comparison with eukaryotic polymers. Symp. Soc. Exp. Biol.

[20] Tolouee M., Alinezhad S., Saberi R., Eslamifar A., Zad S.J., Jaimand K., Taeb J., Rezaee M.B., Kawachi M., Shams-Ghahfarokhi M. (2010). Effect of *Matricaria chamomilla* L. flower essential oil on the growth and ultrastructure of *Aspergillus niger* van Tieghem. Int. J. Food Microbiol.

[21] Shams-Ghahfarokhi M., Goodarzi M., Razzaghi-Abyaneh M., Al-Tiraihi T., Seyedipour G. (2004). Morphological evidences for onion-induced growth inhibition of *Trichophyton rubrum* and *Trichophyton mentagrophytes*. Fitoterapia.

[22] Das K., Tiwari R.K.S., Shrivastava D.K. (2010). Techniques for evaluation of medicinal plant products as antimicrobial agent: Current methods and future trends. J. Med. Plant Res.

